# Gastrointestinal problems in modern wars: clinical features and possible mechanisms

**DOI:** 10.1186/s40779-015-0042-5

**Published:** 2015-06-24

**Authors:** Wei-Feng Wang, Xiao-Xu Guo, Yun-Sheng Yang

**Affiliations:** Department of Gastroenterology and Hepatology, Chinese PLA General Hospital, Beijing, 100853 China

**Keywords:** Military personnel, Veterans, Gulf War syndrome, Functional gastrointestinal disorders, Irritable bowel syndrome, Diarrhea, Dyspepsia

## Abstract

Gastrointestinal problems are common during wars, and they have exerted significant adverse effects on the health of service members involved in warfare. The spectrum of digestive diseases has varied during wars of different eras. At the end of the 20th century, new frontiers of military medical research emerged due to the occurrence of high-tech wars such as the Gulf War and the Kosovo War, in which ground combat was no longer the primary method of field operations. The risk to the military personnel who face trauma has been greatly reduced, but disease and non-battle injuries (DNBIs) such as neuropsychological disorders and digestive diseases seemed to be increased. Data revealed that gastrointestinal symptoms such as constipation, diarrhea, dyspepsia, and noncardiac chest pain are common among military personnel during modern wars. In addition, a large number of deployed soldiers and veterans who participated in recent wars presented with chronic gastrointestinal complaints, which fulfilled with the Rome III criteria for functional gastrointestinal disorders (FGIDs). It was also noted that many veterans who returned from the Gulf War suffered not only from chronic digestive symptoms but also from neuropsychological dysfunction; however, they also showed symptoms of other systems. Presently, this broad range of unexplained symptoms is known as “Gulf War syndrome”. The mechanism that underlies Gulf War syndrome remains unclear, but many factors have been associated with this syndrome such as war trauma, stress, infections, immune dysfunction, radiological factors, anthrax vaccination and so on. Some have questioned if the diagnosis of FGIDs can be reached given the complexity of the military situation. As a result, further studies are needed to elucidate the pathogenesis of gastrointestinal disease among military personnel.

## Introduction

Gastrointestinal problems are common during wars, and they have had a significant adverse impact on the health of service members involved in warfare. Hence, gastroenterology in the armed forces has been one of the hot topics in military medical research. The spectrum of digestive diseases that has been observed during wars has changed with the times. For example, during World War II, the British Navy reported a high incidence of peptic ulcers [1] in soldiers. However, with new innovative drugs that continue to appear on the market, peptic ulcers are no longer a serious problem. Additionally, before the advent of antibiotics, gastrointestinal infections [2] were the predominant type of disease during wars. In recent decades, its harm has been greatly reduced as significant progress has been made in the diagnosis and treatment of these diseases. By the end of 20th century, new frontiers of military medical research had emerged due to the occurrence of high-tech wars such as the Gulf War and the Kosovo War, in which a variety of newly developed high-tech weapons led to the reversal of the traditional opinions on warfare. Ground combat was no longer the leading method of field operations, and as a result, the United States Department of Defense attached more importance to the Navy and Air Force rather than to the maintenance and improvement of the ground forces. Intelligent high-tech weapons have allowed for the most precise bombing in history so that civilian casualties are minimized more than in the past. The risk to the military personnel who face trauma has been greatly reduced, but DNBIs [3–5] such as neuropsychological disorders and digestive diseases seemed to be increased. Hence, in modern wars, more attention has been paid to conditions such as DNBIs compared with other types of war trauma. Shortly after the Gulf War, Gulf War syndrome, a new medical syndrome with an undetermined pathogenesis, was recognized. Chronic digestive problems were cited as one of its most important clinical features [6, 7].

Although much effort has been devoted to chronic digestive problems that occur under military circumstances, they remain an important challenge to the health of soldiers and veterans of modern wars. Here, we aimed to review the common gastrointestinal complaints of soldiers who have participated in modern wars based on a literature search of Pubmed and to discuss the possible pathogenesis of these disorders.

## Review

### Gastrointestinal manifestations in the armed forces

#### Constipation

It has been postulated that constipation has been an important health concern in military operations because recipes in the armed forces have had to be adjusted based on military situations during a war. To facilitate food preservation and transportation, food ingredients and dietary habits are often very different from those in normal daily life, and thus food diversity is not a priority. In addition, in certain situations it would be difficult for military personnel to access fresh vegetables and fruits, which may easily lead to constipation. Some of the data showed that the occurrence of constipation is significantly increased in active duty members of the armed forces. A U.S. study [8] reported that the prevalence of constipation is as high as 34 % in individuals in a combat environment. Similarly, a survey of a small sample of deployed soldiers in Operation Enduring Freedom in Afghanistan [9] showed a high prevalence of constipation. However, contrary to popular opinion, a population-based survey [10] of military servicemen has shown that diarrhea, rather than constipation, is the most common change in bowel habits. We believe that the controversial nature of these studies lies in the different sample sizes [9] and different periods of military engagements. Surveys of a small sample of military personnel or those with specific jobs, such as mariners, revealed a higher incidence [9] of constipation, but the result was just the opposite when a survey was conducted in a large sample or in those with multiple jobs [10].

Because the effect of fiber on the prevention and treatment of constipation is recognized, it is not surprising that more attention has been paid to fiber-rich foods when meals are prepared for soldiers. It seems to be increasingly popular to add fiber to food for not only the general population [11] but also for military servicemen. Increased dietary intake of fiber among military personnel may contribute to the lower-than-expected prevalence of constipation in surveys of large samples.

As is well known, constipation has a significantly negative impact on the quality of life [12] in the general population because it leads to more clinic visits and more absences from work. However, research has been scarce on how constipation impacts the quality of life in military situations. Although limited data are currently available, constipation [8] does not seem to affect a soldier’s typical workday. However, this conclusion should be interpreted with caution due to the small sample size included in this particular study. Only 118 cases were involved, of which 10 were female, and it is well known that constipation is more common in women in the general population. However, the demographic feature of this small sample was much different from that of the general population, which limited the significance of this result.

#### Diarrhea

As mentioned above, one large-scale survey revealed that diarrhea was one of the most frequent complaints [10] in deployed military personnel from the United States. Both acute and chronic diarrhea was prevalent. It was reported [13] that 76.8 % of soldiers in Operation Iraqi Freedom and Operation Enduring Freedom experienced diarrhea. Another study reported [14] an average of 25.2 episodes of diarrhea per month among soldiers. Acute diarrhea is often associated with bacterial infections, but viral infections may also contribute to acute diarrhea. On the contrary, many cases with chronic diarrhea could not be traced to organic causes. Hence, it was often considered to be a functional disease (*i.e.*, functional diarrhea or irritable bowel syndrome (IBS)).

Traveler’s diarrhea is recognized as the most common type [10] of acute diarrhea in military situations. In fact, traveler’s diarrhea is a public health problem not only in soldiers but also in the general population. Theoretically, traveler’s diarrhea may occur at any travel destination, but the most likely places are tropical and subtropical regions, as well as poor and undeveloped areas [15, 16]. People may develop traveler’s diarrhea after ingestion of polluted water or food, but genetic susceptibility also plays an important role in the development of the disease. A global study revealed that the incidence of traveler’s diarrhea in Brazil was 13.6 %, while the incidence of traveler’s diarrhea [16] in Mombasa, Kenya was as high as 54.6 %.

In recent wars, troops from developed countries such as the United States and Britain that maintained high standards of sanitary conditions were deployed to underdeveloped areas or desert areas such as Iraq and Afghanistan. Therefore, they were at high risk for the development of traveler’s diarrhea [14]. In fact, various studies have shown that the incidence of traveler’s diarrhea was high during the Gulf War [17] with occasional outbreaks [18] among the troops.

In regards to the pathogen, the main cause of traveler’s diarrhea is bacterial contamination. *E. coli*, including enterotoxigenic *E. coli* and enteroaggregative *E. coli,* is the most prevalent pathogen. *Campylobacter* and *Salmonella* are also important pathogenic agents [14, 19]. However, sometimes the pathogens cannot be detected by the current culture methods. It will be interesting to know whether the pathogens of traveler’s diarrhea that are identified in troops are similar to those found in the general population. Further studies showed that the types of pathogens varied with different deployment areas and different arms and services. For example, a medical institution located in the United States that serves the Air Force [20] revealed that *Salmonella* and *Campylobacter* were the primary bacterial agents in the development of gastroenteritis among soldiers and their dependents.

#### Dyspepsia

In the 1940s, dyspepsia was the most common digestive complaint among soldiers. In fact, the majority of cases of dyspepsia was caused by peptic ulcers. The prevalence of ulcers in the British army [21] was found to be as high as 55 %. The main reason for the high prevalence of peptic ulcers at that time was likely associated with the lack of effective drugs that were available with the exception of surgery. In addition to ulcer-associated dyspepsia, others were considered to suffer from non-ulcer dyspepsia, which is a type of functional dyspepsia (FD). In 1987, one Italian study [22] of soldiers revealed that dyspepsia was common, as 49 % of soldiers presented with dyspepsia, but only one soldier was confirmed to have a peptic ulcer. Hence, most of the participants in this study were considered to have a diagnosis of functional dyspepsia. After a comparison of these two studies, we found that the incidence of ulcers in the army was significantly decreased. This decrease was attributed to the advent of new innovative drugs for peptic ulcers such as H2 receptor antagonists and proton-pump inhibitors. Contrary to the decreased prevalence of ulcer-related dyspepsia, it seemed that the incidence of FD was increased significantly. However, the reason for this remains unclear.

#### Heartburn

Heartburn is frequently observed in the general population, and GERD is considered to be the major disease [23] that causes heartburn. However, until recent decades, little attention has been paid to the impact of heartburn on military personnel. In 1991, the American Journal of Gastroenterology published a study of Holocaust survivors [24] of World War II from Eastern Europe with a focus on a variety of gastrointestinal symptoms such as heartburn and abdominal pain. The prevalence of heartburn was significantly higher compared with that in the controls who were from the same region but did not endure the extreme mental and physical hardships during World War II. There were similar findings from a recent study of Persian Gulf War veterans, which revealed that heartburn was one of the most common gastrointestinal symptoms [25] that led to endoscopic examination. The major reason for heartburn was esophagitis [25, 26]. Moreover, heartburn of functional origin [24] was also reported.

#### Noncardiac chest pain

Patients with noncardiac chest pain often undergo careful examination of the lungs and the heart, which typically reveals nothing abnormal; hence, chest pain is considered to be of esophageal origin and to be functional. Noncardiac chest pain [27] has also been a common gastrointestinal symptom reported by military personnel during war, but few in-depth studies have been conducted on noncardiac chest pain in the armed forces. A prospective study of 1,935 soldiers during the Iraq War showed that noncardiac chest pain was common, but that it had little effect on the ability of soldiers to return to duty compared with other common symptoms. For patients who present with noncardiac chest pain, empiric treatment remains the first-line management of this condition. Nonsteroidal anti-inflammatory drugs often exhibit therapeutic effects, but only some patients require opioids or antidepressants.

#### Functional gastrointestinal disorders

As is known, FGIDs are highly prevalent in the general population, among which IBS and FD are the most common. It would be interesting to know whether FGIDs are also prevalent among military personnel and what type of FGID is predominant in the armed forces. A population-based survey of the Chinese air force [28] revealed that more than 23 % of air crew and ground personnel reported gastrointestinal symptoms that fulfilled the Rome III criteria for FGIDs. Additionally, a German study reported that approximately 50 % soldiers who sought health care for gastrointestinal problems [29] were diagnosed with FGIDs.

Among a variety of chronic gastrointestinal symptoms studied in military personnel, chronic diarrhea is often found to be the most common ailment [25], followed by dyspepsia, and then heart burn. A study of Persian Gulf War veterans revealed [25] that 63 % of participants presented with diarrhea, and most of the patients with chronic diarrhea had no organic disease. Therefore, we may infer that either functional diarrhea or diarrhea-dominant IBS was the most prevalent disease among FGIDs in this case. It should also be noted that many patients may present with several gastrointestinal symptoms that simultaneously involve both the upper gastrointestinal tract and the lower gastrointestinal tract. Hence, it was not surprising that more overlapping syndromes were observed in military servicemen.

Whether deployment itself could increase the risk of FGIDs remains controversial. It was reported that FGIDs were more prevalent during peacetime and in veterans who returned from deployment, but that they were less prevalent during active duty [29]. On the contrary, a UK study on the Iraq War found that the prevalence of probable IBS was higher in military personnel during deployment than upon their return from Iraq [30].

In the general population, FGIDs such as IBS are more common in women [31] than in men. Whether the same pattern is observed in troops remains unknown. Thus far, no specific study has focused on gender predominance in military personnel with FGIDs. One study reported that no gender differences were found in the majority of 50 symptoms [32, 33] that were presented by military personnel. Hence, this study indicated that no female predominance exists in FGIDs in military circumstances.

#### The Gulf War syndrome

The Gulf War was a U.S.-led war against Iraq in the 1990s in which several Western countries participated. It began with long-distance bombardment, and ground combat was supplementary. At the same time, many new high-tech weapons were applied during this war, including depleted uranium munitions, armoring and laser-guided missiles. It represented the beginning of a new era of modern wars, which led to huge changes in the social, economic and political issues all over the world and brought about new frontiers in military medical research.

As the Gulf War progressed, more and more veterans reported unexplained symptoms, which attracted the attention of health professionals. Western governments launched related studies, and an official committee was organized and supported by the United States government. This committee performed systematic reviews on the research and compiled an official report on this issue. Finally, Gulf War syndrome, also known as Gulf War illness, was recognized. At present, most people support the existence of Gulf War syndrome although some continue to doubt whether this syndrome should be considered an independent disease. There is no consistent definition of Gulf War syndrome, but evidence indicates that multiple organ systems are involved [34, 35], such as the nervous system, digestive system, and respiratory system, in which chronic gastrointestinal symptoms are considered to be one of the main features. The patients with Gulf War syndrome often exhibit abdominal pain, abdominal discomfort, diarrhea, or dyspepsia, which are compatible with the diagnosis of FGIDs.

### Possible mechanism that underlies digestive problems

Military exercises and war are the most common forms of employment of military resources. With the exception of conventional weapons, various chemical, biological and nuclear weapons have threatened members of the armed forces who have been involved in modern wars. In addition, members of the armed forces are often deployed to aid in anti-terrorism operations and disaster relief. Therefore, soldiers confront not only physical trauma but also other hazardous factors such as biological or nuclear threats, earthquakes, and floods. Considering that chronic gastrointestinal symptoms were common among those deployed soldiers, it is worthwhile to investigate the role of the above-mentioned factors in the pathogenesis of chronic gastrointestinal diseases.

#### Trauma

Trauma refers to all forms of injuries that soldiers may experience when they serve in combat. During the era of conventional wars, trauma attracted a large amount of attention for centuries as far as military medicine was concerned. In modern wars, the risk for war trauma has decreased significantly among soldiers who are deployed to war zones compared with the risk among soldiers of previous conventional wars. Hence, less importance has been attributed to war trauma by the military healthcare system. Because high-tech weapons are widely applied and many may possess great explosive force and cause more severe injuries, war trauma is still an important concern of military medicine. It is known that severe burning, multiple fractures, abdominal trauma and other injuries will always lead to the disturbance of multiple systems. Therefore, dysfunction of the digestive system may also occur.

#### Stress and post-traumatic stress disorder (PTSD)

Stress is frequently encountered during military operations, which may significantly impact the combat capability of deployed soldiers. In addition, PTSD is often experienced during war [36, 37]. This type of mental disorder always occurs after an individual experiences a severe injury, threats of death, or even after witnessing death. These patients may manifest as if they are constantly immersed in trauma-related situations, and they may try to avoid trauma-related persons or things, or may have signs of hypervigilance and irritability. During the Gulf War, stress was found to be prevalent in military personnel. Many studies suggested that stress [35] was an important factor that gave rise to Gulf War syndrome. Specifically, factors such as hearing about chemical weapon threats, participation in battles, and the experience of death around them, were found to be related to the onset of Gulf War syndrome.

Many patients with Gulf War syndrome were also found to suffer from depression. It is well known that psychosocial factors such as depression and anxiety play some role in the pathogenesis of functional gastrointestinal disease [38, 39]. Therefore, it is not surprising that these patients often complained of chronic digestive symptoms [40]. Actually, during World War II, much attention was paid to functional gastrointestinal disorders [41, 42] among military personnel. At that time, investigators established the concept that FGIDs may be psychogenic. Until now, we are still exploring how psychosocial factors play a role in the pathogenesis of functional gastrointestinal disorders.

As far as the military situation is concerned, soldiers with PTSD should be distinguished from those with traumatic brain injury [43] without obvious physical trauma. In modern warfare, great progress has been made in high-tech protective measures such as body armor and helmets; hence, the incidence of traumatic brain injury has decreased significantly. However, it was found that a powerful explosion may lead to traumatic brain injury without obvious physical trauma, which may result in mental and cognitive abnormalities, similar to PTSD. Researchers in the United States noticed this type of injury during the wars in Iraq and Afghanistan, and believed that they played an important role in neurological and mental abnormalities among active-duty military personnel.

#### Infections

Infections of the gastrointestinal tract often lead to acute onset of nausea, vomiting and diarrhea. However, several months after effective treatment of gastrointestinal infections, some of these gastrointestinal symptoms may persist, which means they may be FGIDs. It has been reported that gastrointestinal infections may result in post-infectious FGIDs, especially IBS [44, 45]. An epidemiological study revealed that after acute gastroenteritis, approximately 4 %-26 % of individuals may develop IBS [44]. Additionally, up to 30 % of patients with IBS [46, 47] were considered to have post-infectious IBS. As stated above, traveler’s diarrhea is common in modern wars. Evidence has shown that military personnel with traveler’s diarrhea are at a higher risk for the development of FGIDs such as IBS [48].

In regards to the pathogens, bacterial infections are the main factors that cause post-infectious IBS. It was also found that protozoa such as *Giardia lamblia* [49] may contribute to post-infectious IBS. Nevertheless, the mechanism that underlies post-infectious IBS remains unclear [50]. As low-grade intestinal inflammation is often observed in patients with IBS, it is probable that the existence of low-grade inflammation after acute infection accounts for the presence of chronic gastrointestinal symptoms.

#### Radiological, biological and chemical factors

Thus far, three types of weapons of mass destruction (WMDs) (*i.e.*, nuclear weapons, chemical weapons and biological weapons), have been developed. Although there are international treaties that prohibit the development, stockpiling and use of WMDs, the ghost of WMDs has still occasionally appeared during wars in recent decades. For example, the United States bombed Japan with nuclear weapons [51] during World War II. Additionally, data revealed that Unit 731 of the Imperial Japanese Army, known as the Epidemic Prevention and Water Purification Department during World War II, conducted germ warfare attacks [52] in China. The Gulf War was launched by the United States and its allies based on the suspicion that the Iraqi government had its WMD programs. Finally, no evidence of WMDs was found in Iraq after the war. However, during that war, depleted uranium bombs were applied which contained radioactive uranium. Some believed that depleted uranium bombs were not nuclear weapons, and thus the application of them was not restricted by international treaties on WMDs. However, depleted uranium bombs can produce radioactive gas and lead to a devastating impact after the explosion. The gas can be inhaled or enter directly into a wound. Furthermore, it can contaminate the soil and water where the explosion occurred. Unfortunately, data were limited on the hazards of depleted uranium bombs and how they might impact the environment and human health. Thus far, the short-term and long-term harms caused by depleted uranium bombs remain unclear. Recently, studies have supported the concept that Gulf War syndrome [53, 54] was related to the use of depleted uranium bombs.

Before the Gulf War, Western countries worried that the Iraqi army may apply chemical or biological weapons once the war began. Therefore, Western countries took a series of prophylactic measures such as vaccination against chemical and biological weapons as they prepared for the Gulf War. Contrary to expectation, data revealed that these prophylactic measures themselves may have been harmful to those who were vaccinated. During the Gulf War, the authorities in command of the military forces of the United States approved the Anthrax Vaccine Immunization Program out of fear that the Iraqi government may have used anthrax as a biological weapon against Western troops. At that time, the United States Department of Defense claimed that the application of the anthrax vaccine was safe although none of the combatants of the war launched an anthrax attack. When systematic studies were conducted among veterans who returned from the Gulf War, an association was found between large-scale applications of anthrax vaccines in the United States Army and Gulf War syndrome [55, 56].

In addition, it was suspected that Syria and Libya had developed biochemical weapons for decades. Recently, international society paid close attention to a sarin attack [57] in Syria. Sarin, a highly toxic nerve agent, was prohibited by international treaty, but unfortunately, it was used in the Syrian War. However, the United Nations failed to determine which side of the war should be responsible for the attack. The sarin attack in Syria resulted in a large number of civilian casualties via damage to the human nervous system. Previous studies have shown that besides the nervous system, other systems may also be impaired by sarin. Among the victims who survived, some had PTSD [58] while some presented with chronic systemic manifestations in addition to neurological problems. The soldiers deployed to these areas may have been exposed to these biochemical weapons without their knowledge. Pyridostigmine bromide (PB) is a cholinesterase inhibitor [59] that was used by American troops as preventive measure against nerve gas such as sarin during the Gulf War. Some studies also suggested that pyridostigmine bromide may have impaired the health of soldiers who ingested it and that it may have subsequently contributed to the development of Gulf War syndrome.

Most of the countries in the Middle East where the Gulf War occurred were rich in oil reserves. Therefore, oil fields became the focus of bombing during the war. A large number of oil wells burned in fires that lasted for several months. The burning of crude oil produced a large amount of smoke while unburnt oil fell back to the earth. Therefore, the air, water and the soil were severely polluted. This event would undoubtedly cause serious damage to the health of local residents and military service members. Many studies have indicated that oil well fires acted as important factors that may have led to Gulf War syndrome [60, 61].

The reason why chronic digestive symptoms were common among soldiers who were involved in the Gulf War remains unclear. Biological, chemical and radiological factors were linked to Gulf War syndrome. As chronic gastrointestinal symptoms were one of the main features of Gulf War syndrome, these factors may underlie the gastrointestinal manifestations reported by deployed military personnel. How these factors affect the digestive system remain to be elucidated. Furthermore, it should be noted that in modern wars, the risk of exposure to radiological, biological or chemical factors is high but is sometimes concealed. Certain manifestations may develop among deployed soldiers due to exposure to these hazardous factors without knowing it. Thus, it became questionable whether the diagnosis of FGIDs could be reached if the complexity of the military situation in modern wars is considered.

#### Immune dysfunction

It is widely accepted that stress [62] and intense training [63] may have an adverse impact on the function of the immune system. Because military personnel are often subjected to intense physical activity and psychological stress during war or military exercises, it is natural that researchers linked the abnormality of the immune system with the diseases that accompany war or military exercises [64–66]. Thus far, many studies on deployed troops or veterans have explored the humoral immunological status as well as cellular immunity. Components of the immune system such as natural killer cells, T lymphocytes, interleukins and interferon have been widely investigated. Evidence supported altered immune function was generated by military circumstances, especially during the Gulf War [67]. Moreover, Gulf War syndrome was even proposed to be an autoimmune disease [68] that was initiated by the use of a prophylactic nerve agent and adrenergic agents.

However, whether Gulf War syndrome can be attributed to immune dysfunction remains controversial [69]. For example, a Danish Gulf War study [70] reported that no long-term changes in natural killer cell activity or in the production of several cytokines such as interleukins and interferon were present. Moreover, the authors noted that cryopreservation was an important factor that exerted a direct impact on the status of natural killer cells and T lymphocytes, which subsequently may have influenced the soundness of the results. Additionally, one study by the Department of Veterans Affairs Medical Center in Birmingham [71] revealed no abnormalities in the *in vitro* immune responses after a comparison of symptomatic veterans who returned from the Gulf War with the other two control groups, which included asymptomatic veterans who once participated in the Gulf War and non-Gulf War veterans with a disability. Thus, compelling evidence in regards to the role of abnormal immunological changes in individuals with Gulf War syndrome is lacking.

Recently a study in Singapore found that combat-training induced immune activation [72], which might be associated with gastrointestinal symptoms. To date, efforts in this field remain scarce. Further studies are needed to elucidate the relationship between immune activation and gastrointestinal symptoms.

## Conclusion

Digestive symptoms such as constipation, diarrhea, dyspepsia, heartburn, and noncardiac chest pain are common among military personnel in modern wars. Recent data revealed that a large number of deployed soldiers and veterans who participated in these wars often present with chronic gastrointestinal complaints that are consistent with the criteria for FGIDs. It was also noted that many veterans who returned from the Gulf War suffered not only from chronic digestive symptoms but also symptoms of other systems. This broad range of unexplained symptoms is known as Gulf War syndrome. Presently, the mechanism that underlies Gulf War syndrome remains unclear. Many factors have been associated with this syndrome such as war trauma, stress, infections, immune dysfunction, radiological factors, and anthrax vaccination. As is known, a formal diagnosis of FGIDs requires the exclusion of organic diseases as well as the exclusion of known pathogenic agents. The existence of these biological, chemical and radiological factors during modern wars has undermined the confidence of medical staff to reach a diagnosis of FGIDs in military personnel, although these chronic gastrointestinal symptoms are compatible with the criteria for FGIDs. Therefore, further studies are needed to elucidate this issue.

## Abbreviations

DNBIs: Disease and non-battle injuries
FD: Functional dyspepsia
FGIDs: Functional gastrointestinal disorders
IBS: Irritable bowel syndrome
PB: Pyridostigmine bromide
PTSD: Post-traumatic stress disorder
WMDs: Weapons of mass destruction
